# Preclinical assesement of survivin and XIAP as prognostic biomarkers and therapeutic targets in gastroenteropancreatic neuroendocrine neoplasia

**DOI:** 10.18632/oncotarget.14207

**Published:** 2016-12-26

**Authors:** Levent Dizdar, Kira A. Oesterwind, Jasmin C. Riemer, Thomas A. Werner, Sabrina Mersch, Birte Möhlendick, Sina C. Schütte, Pablo E. Verde, Katharina Raba, Stefan A. Topp, Nikolas H. Stoecklein, Irene Esposito, Wolfram T. Knoefel, Andreas Krieg

**Affiliations:** ^1^ Department of Surgery (A), Heinrich-Heine-University and University Hospital Duesseldorf, Moorenstr. 5, 40225 Duesseldorf, Germany; ^2^ Institute of Pathology, Heinrich-Heine-University and University Hospital Duesseldorf, Moorenstr. 5, 40225 Duesseldorf, Germany; ^3^ Coordination Centre for Clinical Trials, Heinrich-Heine-University and University Hospital Duesseldorf, Moorenstr. 5, 40225 Duesseldorf, Germany; ^4^ Institute for Transplantation Diagnostics and Cell Therapeutics, Heinrich-Heine-University and University Hospital Duesseldorf, Moorenstr. 5, 40225, Duesseldorf, Germany

**Keywords:** survivin, XIAP, GEP-NEN, neuroendocrine neoplasia, inhibitor of apoptosis protein

## Abstract

Gastroenteropancreatic neuroendocrine neoplasms (GEP-NEN) represent a rare and heterogenous tumor entity. Importantly, the highly proliferative subgroup of neuroendocrine carcinoma (GEP-NEC) is characterized by high resistance to conventional chemotherapy. Consequently, there is an urgent need to identify novel therapeutic targets, especially for GEP-NEC. Thus, we focused on Inhibitor of apoptosis protein (IAP) family members survivin and XIAP that orchestrate inhibition of apoptosis, induce resistance against chemotherapeutics and facilitate tumor metastasis. Copy number gains (CNGs) could be detected by microarray comparative genomic hybridization for survivin and XIAP in 60 % and 26.7 % of all GEP-NENs, respectively. Immunohistochemical staining of tissue specimens from 77 consecutive patients with GEP-NEN demonstrated increased survivin protein expression levels in tissue specimens of highly proliferative GEP-NEC or GEP-NEN located in the stomach and colon. In contrast, XIAP overexpression was associated with advanced tumor stages. Knockdown of survivin and XIAP markedly reduced cell proliferation and tumor growth. In vitro, YM155 induced apoptotic cell death accompanied by a reduction in cell proliferation and inhibited GEP-NEC xenograft growth. Taken together, our data provide evidence for a biological relevance of these IAPs in GEP-NEN and support a potential role of survivin as therapeutic target especially in the subgroup of aggressive GEP-NEC.

## INTRODUCTION

Gastroenteropancreatic Neuroendocrine Neoplasms (GEP-NEN), characterized by the expression of general neuroendocrine markers such as chromogranin A (CgA) or synaptophysin, represent a rare and heterogenous tumor entity with an estimated annual incidence of up to 2.51/100.000 [1–3]. According to the revised WHO classification published in 2010, GEP-NEN are currently classified on a proliferation based categorization into well (G1) or moderately differentiated (G2) neuroendocrine tumors (NET) and the poorly differentiated (G3) large cell or small cell type neuroendocrine carcinomas [4].

To date, complete surgical resection using standard oncological principles remains to be the first line therapy for patients with localized or limited disease [5]. Unfortunately, about 70% of patients with newly diagnosed GEP-NEN present with a metastasized disease [3]. For patients with distant metastasis, optimized interdisciplinary treatment approaches such as surgical debulking, interventional embolization techniques, radiofrequency ablation, chemotherapy or nuclear medicine therapies can be considered and are sometimes beneficial [6]. With current chemotherapeutic agents, including streptozotocin, 5-fluorouracil (5-FU), cisplatin in combination with etoposide, or small molecules targeting growth factor receptors, tyrosine kinases, mTOR signaling components and somatostatin receptors prolonged survival can be observed only in a subset of GEP-NEN patients [7, 8]. The first line therapy for GEP-NEC using cisplatin and etoposide achieves only a 50 % response rate and a second line therapy has not been established yet [9, 10]. Consequently, there is an urgent need to identify novel therapeutic targets especially for the group of highly aggressive GEP-NEC.

One of the cancer hallmarks is a disturbed balance between cell death and survival [11], which is facilitated by an altered expression of proteins being involved in the regulation of apoptosis. In this context, a group of anti-apoptotic BIR (baculovirus IAP repeat)-domain containing proteins referred to as the inhibitor of apoptosis protein (IAP) family has attracted considerable attention during the last decades [12]. Among them, survivin/BIRC5 and X-linked inhibitor of apoptosis protein XIAP/BIRC4 are the most extensively investigated members. Functionally, survivin stabilizes XIAP through direct interaction, thus preventing XIAP polyubiquitination and subsequent proteosomal degradation [13]. This complex synergistically inhibits caspases and activates NF-κB signaling via recruitment of the adaptor molecule TAB1, resulting in a consecutive activation of TAK1 and IKKs, which leads to phosphorylation-induced IκBα degradation and nuclear p50/p65 translocation [13–15]. Importantly, NF-κB activation via the survivin-XIAP complex has been shown to induce tumor cell invasion and formation of metastasis [15]. In addition, survivin participates as a chromosomal passenger protein in the control of mitosis [16, 17].

Since their identification, many studies in almost every human tumor including endocrine neoplasms demonstrated an overexpression of survivin and XIAP [18]. In addition, most of these studies have identified survivin as risk factor for poor prognosis and tumor recurrence which has been recently supported by several meta-analysis [19, 20]. Consequently, efforts have been intensified in the development of small molecule IAP-antagonists that selectively target cancer cells and prevent normal cells from programmed cell death. Among the survivin small molecule antagonists, YM155 (Sepantronium bromide) and tetra-O-methyl nordihydroguaiaretic acid (M4N; Terameprecol; EM-1421) demonstrated preclinical acitivity in several tumor entities with a favorable safety profile in phase I studies [21–24]. However, phase II clinical trials revealed limited activity as monotherapy and suggest the application of YM155 as part of a combination therapy with other chemotherapeutic agents [25–27]. Mechanistically, both YM155 and M4N act as transcriptional repressors by antagonizing Sp1-dependent survivin promotor activation [28]. In contrast, small molecules that mimic the endogenous IAP-inhibitor smac antagonize XIAP mediated inhibition of caspases by directly competing for binding or by degrading IAP-members c-IAP1 and 2 [29].

While the expression and potential therapeutic role of IAPs has been demonstrated in several tumor entities, the role of survivin and XIAP in the biology of GEP-NEN remains to be elucidated. Previously, by comparative genomic hybridization (CGH) Tönnies and colleagues detected gains at chromosome 17q in 57 % of midgut NEN [30]. Against the backdrop of the known BIRC5 genetic region on chromosome 17q and the observation that immunhistochemically detected expression of survivin was associated with a poor prognosis in a subset of GEP-NEN [31], these studies suggest that survivin might be a viable target in the therapy of GEP-NEN. Considering these observations and the functional linkage between survivin and XIAP, our current study sought to evaluate both survivin and XIAP as prognostic biomarkers according to the REporting recommendations for tumor MARKer prognostic studies (REMARK) and to further assess their potential as therapeutic targets in GEP-NEN [32].

## RESULTS

### Copy number alteration and expression of survivin and XIAP in GEP-NEN

**Figure 1:**
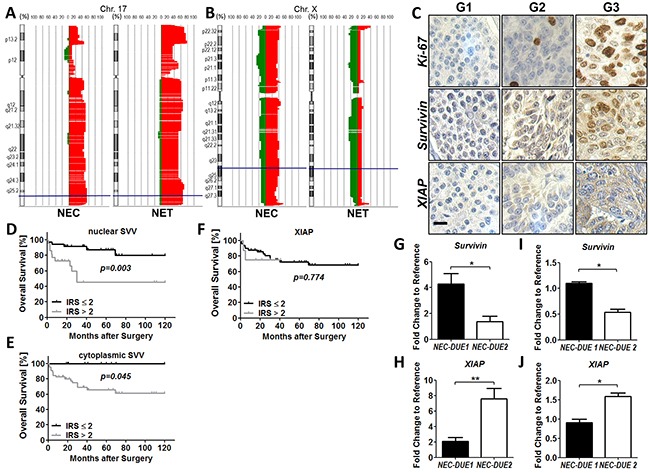
Copy number alterations (CNA) and expression levels of survivin and XIAP in GEP-NEN Copy number alterations on chromosome 17 and X were evaluated by aCGH analysis. Filled areas denote gains (right red area) or losses (left green area) and X-axis indicate the percentage of specimens with gains or losses. **A.** High frequencies of CNAs were observed at the survivin locus (blue lines) in G1/G2 GEP-NET and G3 GEP-NEC. **B.** XIAP CNAs (blue lines) affect more frequently highly aggressive GEP-NEC. **C.** Representative images of Ki-67 (top), survivin (middle) and XIAP (bottom) in low proliferative G1 (Ki-67-index: ≤2%), G2 (Ki-67-index: 2-20%) and highly proliferative G3 (Ki-67: >20%) GEP-NEN. Images were captured at 400 × magnification and scale bar indicates 10 µm. Kaplan-Meier curves represent the prognostic value of nuclear and cytoplasmic survivin **D.** and **E.** and XIAP **F, G.** Data from qRT-PCR shows markedly expressed survivin in GEP-NEC cell line NEC-DUE1. H, NEC-DUE2 cells expressed higher levels of XIAP RNA when compared to NEC-DUE1 cells. **I.** and **J.** Western blot analysis confirmed the differential expression of survivin (I) and XIAP (J) in NEC cell lines. Values are expressed in means ± SEM of three independent experiments. Statistical significance was calculated by Mann-Whitney U test (**indicates a p-value ≤0.01; *indicates a p-value ≤0.05).

Previously, a study using conventional comparative genomic hybridization (CGH) method demonstrated frequent copy number gains (CNGs) at chromosome 17q in midgut GEP-NEN [30]. Since the genetic locus encoding survivin is located on chromosome 17q25 we took advantage of the high-resolution array CGH (aCGH) and analyzed the copy number alterations (CNAs) at chromosome 17q in a set of 45 GEP-NENs from distinct anatomical sites and different types of differentiation (Figure 1A). Given that survivin functionally interacts with XIAP, we additionally analyzed CNAs at the X-chromosome (Figure 1B). We detected CNGs for survivin and XIAP in a total number of 27 (60 %) and 12 (26.7 %) GEP-NENs, respectively. Whereas no difference became evident in the frequence of gains located within the chromosaml region of survivin when comparing G1/G2 NET with G3 NEC (62.5% versus 57.1%) (Figure 1A), we observed a higher percentage of XIAP CNGs in the biologically aggressive group of NECs (NET : NEC = 16.7% : 38.1%) (Figure 1B).

Next, we investigated if overall survival was related to CNGs of survivin or XIAP in GEP-NENs by creating Kaplan-Meier curves. Although no statistically significant difference in survival proportions became obvious when comparing patients with and without a CNG of survivin and/or XIAP, patients with XIAP CNG showed a tendency to a less favorable outcome (p = 0.167) (Supplementary Figure 1A and 1B). In addition, we did not find correlations between survivin or XIAP gene amplification status and any of the patients’ clinicopathological parameters.

To further investigate whether CNAs affecting the chromosomal loci of survivin and XIAP correlated with the protein expression levels of both IAPs, we constructed a tissue microarray (TMA) of surgically resected tissue specimens from patients with GEP-NEN. After excluding patients from whom there was no tissue of the primary tumor available, we were able to include a total number of 77 patients that underwent surgery for primary GEP-NEN at our department (including the 45 patients from aCGH analysis). The main characteristics of these patients are summarized in Supplementary Table 1. Immunohistochemical staining of TMAs demonstrated a cytoplasmic and nuclear expression for survivin in GEP-NEN (Figure 1C). In contrast, XIAP exhibited a predominately cytoplasmic localization. However, when comparing protein levels of survivin or XIAP with the corresponding CNAs detected by aCGH, no significant correlation became evident (Supplementary Figure 1C-1E).

Next, we analyzed whether protein levels of XIAP and survivin correlated with clinicopathological parameters (Table 1). Whereas XIAP expression was not associated with the proliferation based categorization of GEP-NEN, nuclear and cytoplasmic survivin exhibited higher expression levels in the group of highly proliferative G3 tumors. Notably, an increased nuclear and cytoplasmic survivin expression became obvious in the group of GEP-NEN located in the stomach and colon, respectively. In contrast to survivin, XIAP overexpression was associated with advanced tumor stages. For the other clinicopathological parameters we could not observe a significant correlation with survivin or XIAP (Table 1).

**Table 1:** Correlation between survivin or XIAP expression and clinicopathological factors in GEP-NEN

Variable	Survivin (nuclear)		*p*-value	Survivin (cytoplasmic)		*p*-value	XIAP		*p*-value
	Low (IRS ≤ 2)	High (IRS > 2)		Low (IRS ≤ 2)	High (IRS > 2)		Low (IRS ≤ 2)	High (IRS > 2)	
*Sex*									
Male	22	16	0.74	9	29	0.54	32	6	0.47
Female	24	15		7	32		35	4	
*T-category*									
T1+T2	22	10	0.29	8	24	0.65	31	1	**0.03**
T3+T4	22	17		8	31		31	8	
*Lymph node metastasis*									
Negative	20	13	0.94	6	27	0.45	29	4	0.93
Positive	24	15		10	29		34	5	
*Distant metastasis*									
Negative	27	23	0.16	8	42	0.16	42	8	0.28
Positive	19	8		8	19		25	2	
*Grading*									
G1+G2	43	14	**< 0.001**	15	42	**0.04**	52	5	0.06
G3	3	17		1	19		15	5	
*Resection margins*									
Negative	38	27	0.59	12	53	0.24	56	9	0.60
Positive	8	4		4	8		11	1	
*Localisation*									
Pancreas	26	9	**< 0.001**	7	28	**0.01**	31	4	0.23
Stomach	3	6		2	7		7	2	
Small intestine	13	2		7	8		15	0	
Colon	4	14		0	18		14	4	

To explore the role of survivin and XIAP as prognostic markers in GEP-NEN we performed Kaplan-Meier analyses and estimated differences in 10-year overall survival by using log-rank test as well as a Cox proportional Hazard model. Accordingly, high nuclear and cytoplasmic survivin expression was significantly associated with poor outcome (Figure 1D and 1E) but not XIAP (Figure 1F). However, multivariate analysis retained only G3 grading and the presence of lymph node metastasis as independent prognostic factors that were strongly associated with a shorter overall survival in GEP-NEN. The complete findings from univariate and multivariate survival analysis are summarized in (Supplementary Table 2 and 3).

Since our imunohistochemical data revealed first evidence for a biological role of survivin and XIAP in GEP-NEN tumor biology, we next evaluated the expression levels of both IAP members in our recently established NEC cell lines (NEC-DUE1 and NEC-DUE2). Whereas in NEC-DUE1 an increased expression of survivin on RNA level became detectable, NEC-DUE2 cells exhibited higher expression levels of XIAP as revealed by quantitative RT-PCR (Figure 1G and 1H). Importantly, these data were further confirmed on protein level by using Western blot analysis (Figure 1I and 1J).

### Survivin and XIAP knockdown impairs GEP-NEC proliferation and tumor growth

**Figure 2:**
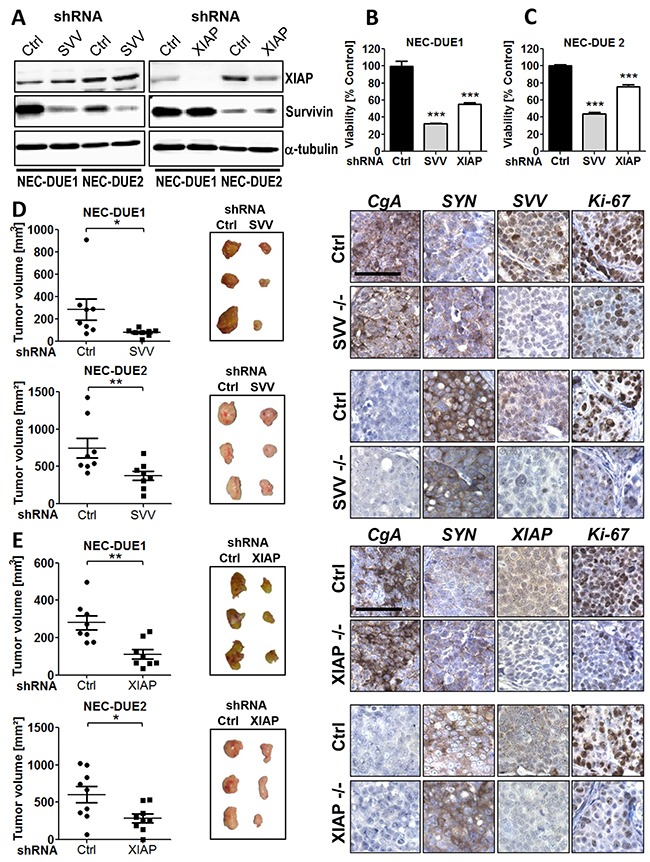
Knockdown of survivin or XIAP impairs growth of GEP-NEC cells both in vitro and in vivo **A.** Western blot confirmed an effective knockdown of survivin or XIAP after transduction of NEC-DUE1 and -DUE2 cells with the respective gene-specific shRNAs. Alpha-tubulin served as loading control. Lentiviral transduction of NEC-DUE1 **B.** and NEC-DUE2 **C.** with indicated shRNAs targeting survivin (SVV), XIAP reduced cell viability in vitro. A nonspecific shRNA served as control (Ctrl). Freshly transduced NEC cell lines were seeded at a density of 2×104 cells per well in 96 well plates. After 7 days cell viability was measured by performing MTS assays. Values are expressed in means ± SEM. Statistical significance was calculated by two-tailed nonparametric Mann-Whitney test (***indicates a p-value <0.001). **D.** and **E.** Gene-silencing of Survivin and XIAP abrogates tumor growth in a xenograft mouse model of GEP-NEC. 1×106 NEC-DUE1or NEC-DUE2 (D) survivin (SVV) or (E) XIAP knockdown cells were subcutaneously injected into the left flank region of 6-8-week-old NOD-Scid IL2rgammanull mice. NEC cells transduced with unspecific shRNA control (Ctrl) were injected in the other side to serve as their own control. After 3-5 weeks mice were sacrified and tumors were resected to measure the volume and weight. Sections of FFPE tumor specimens were immunohistochemically assessed for the expression of chromogranin A (CgA) and synaptophysin (SYN) as indicated. Staining with antibodies raised against human survivin (SVV) or XIAP confirmed a gene-specific knockdown. Proliferation was assessed by Ki-67 staining. Images were captured at 400 × magnification and scale bar indicates 25 µm. Values are expressed in means ± SEM of at least 8 tumors per shRNA transduced and injected cell line. Statistical significance was calculated by Wilcoxon matched pairs test (**indicates a p-value ≤0.01; *indicates a p-value ≤0.05).

To elucidate the biological relevance of survivin and XIAP in promoting GEP-NEC tumor growth, we performed both *in vitro* and *in vivo* loss of function experiments using a shRNA approach. Therefore, we lentivirally transduced NEC cell lines using GIPZ shRNA constructs specifically targeting human survivin and XIAP, respectively. In addition, a non-targeting lentiviral shRNA construct served as negative control. Western blot analysis confirmed a marked knockdown of survivin and XIAP, respectively (Figure 2A). Importantly, expression levels of survivin in XIAP knockdown cells remained unchanged and vice versa. To explore the effect of a targeted knockdown in survivin or XIAP deficient cells *in vitro*, we quantified cell viability of freshly transduced NEC cell lines after growing for a period of 7 days by performing MTS assays. Interestingly, survivin knockdown reduced cell viability in NEC-DUE1 cells up to 32.4% (p < 0.001) and in NEC-DUE2 cells up to 43.9% (p < 0.001) when compared to control cells. Similar results were observed after XIAP knockdown in NEC-DUE1 (55.4 %; p < 0.001) and NEC-DUE2 (75.7 %; p < 0.001) cells (Figure 2B and 2C). Reduction in cell viability was accompanied by caspase-3 activation, with the most pronounced effect in XIAP knockdown cells when compared to control cells (Supplementary Figure 2A and 2B).

Based on our knockdown cell culture experiments, suggesting a biological role for both IAPs survivin and XIAP in GEP-NEN, we aimed to confirm these observations *in vivo* by using a NEC xenograft mouse model. Therefore, we injected survivin or XIAP knockdown NEC cells into the flank of immunocompromized mice. In addition, control cells were injected into the oposite flank. Consistent with our *in vitro* data, targeted knockdown of survivin or XIAP markedly suppressed *in vivo* tumor growth of both NEC cell lines. This was characterized by a reduced average tumor volume in the survivin knockdown tumors when compared with control tumors at study termination [NEC-DUE1: 78.3 mm^3^ (± 11.68) versus 283.4 mm^3^ (± 95.43), p = 0.023; NEC-DUE2: 375.6 mm^3^ (± 62.65) versus 745.0 mm^3^ (± 131), p = 0.008] (Figure 2D). Moreover, survivin knockdown was associated with a decreased tumor weight when compared with controls [NEC-DUE1: 0.05 g (± 0.01) versus 0.15 g (± 0.02), p = 0.014; NEC-DUE2: 0.34 g (± 0.05) versus 0.58 g (± 0.09), p = 0.016) (Supplementary Figure 2C and 2D). Similar results were obtained for XIAP-deficient NEC cells that demonstrated an impaired average tumor growth [NEC-DUE1: 111.1 mm^3^ (± 25.72) versus control: 279.8 mm^3^ (± 38.5), (p = 0.008) and NEC-DUE2: 284.9 mm^3^ (± 57.95) versus control 603.9 mm^3^ (± 109.8), p = 0.027)] and reduced average tumor weight (NEC-DUE1: 0.06 g (± 0.01) versus control 0.09 g (± 0.01), (p = 0.023) and NEC-DUE2: 0.31 g (± 0.06) versus control 0.52 g (± 0.07), p = 0.039) (Figure 2E and Supplementary Figure 2E and 2F).

To confirm the stable knockdown of NEC cell lines within the tumors, tissue sections from tumors of each experimental group were immunohistochemically stained with antibodies raised against human survivin and XIAP, respectively. As expected, tumors derived from gene-specific knockdown cell lines exhibited a decreased expression of the respective target proteins survivin or XIAP (Figure 2D and 2E). Moreover, all tumors retained the typical expression of general neuroendocrine markers CgA or synaptophysin regardless of their survivin or XIAP expression status. In addition, knockdown of survivin or XIAP was accompanied by a pronounced decrease in tumor cell proliferation of NEC tumors as assesed by Ki-67 staining (Figure 2D and 2E).

### *In vitro* effects of survivin and XIAP small molecule antagonists

**Figure 3:**
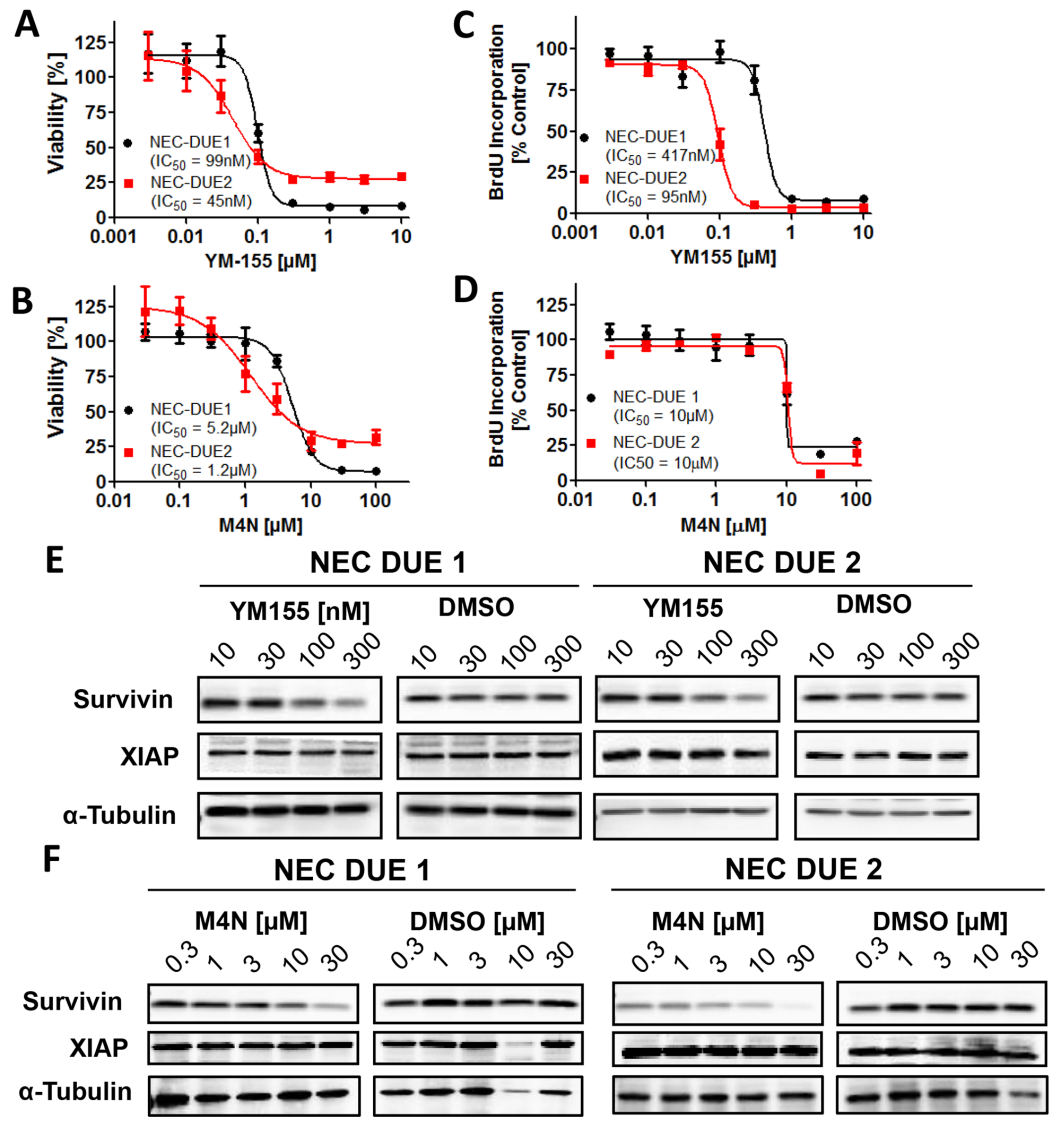
Small molecule survivin antagonists YM155 and M4N reduce cell viability and impair cell proliferation in GEP-NEC cell lines A-D, 5×104 cells were seeded per well in 96 well plates and incubated for 4 days with increasing concentrations of YM155 **A.** and **C.** or M4N **B.** and **D.** as indicated. DMSO served as vehicle control at equimolar concentrations. A and B, cell viability and C and D, proliferation assays were performed in triplicates. Incubation of NEC-DUE1 and -DUE2 cells for 24 hours with increasing concentrations of YM155 **E.** and M4N **F.** induced a dose dependent decrease in survivin protein levels. XIAP protein levels remained unchanged.

The observation that survivin and XIAP knockdown impairs tumor growth of NEC cell lines tempted us to investigate the growth-inhibitory and pro-apoptotic effects of IAP antagonizing compounds on NEC-DUE cell lines. To investigate if survivin antagonists YM155 (Sepantronium Bromide) and M4N (Tetra-O-methyl nordihydroguaiaretic acid) affect cell viability of NEC cell lines, we incubated NEC-DUE1 and -2 cells with increasing concentrations of YM155 and M4N, respectively. Both YM155 and M4N induced a dose dependent decrease in cell viability of NEC-DUE1 and NEC-DUE2 cells with an IC_50_ of 99 nM and 45 nM for YM155 and 5.2 µM and 1.2 µM for M4N (Figure 3A and 3B). Of note, NEC-DUE1 cells exhibiting increased survivin mRNA and protein expression levels, showed higher IC_50_ values upon treatment with both antagonizing survivin compounds. Compatible with the effects of YM155 on cell viability, proliferation measured by BrdU (Bromodeoxyurdine) incorporation was 4 fold decreased in NEC-DUE2 cells when compared to NEC-DUE1 cells (Figure 3C). In contrast, M4N mediated inhibition of BrdU incorporation was comparable among the NEC cell lines (Figure 3D). Importantly, for both compounds and NEC cell lines this effect was accompanied by a dose dependent decrease in survivin but not in XIAP protein levels (Figure 3E and 3F).

Since XIAP knockdown negatively impaired tumor growth of NEC-DUE cell lines *in vivo*, we took advantage of the well characterized smac mimetics Birinapant and GDC-0152 that have been demonstrated to selectively antagonize IAPs. However, both IAP-antagonsits failed to significantly reduce cell viability (Supplementary Figure 3A and 3B).

**Figure 4:**
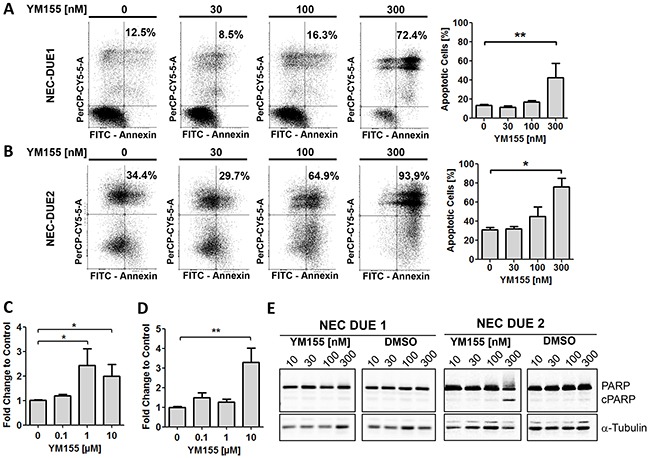
YM155 induces apoptotic cell death in GEP-NEC cell lines Annexin-V/PI staining and FACS analysis of NEC-DUE1 **A.** or -DUE2 **B.** cells revealed a dose dependent increase of annexin positive apoptotic cells when incubated with YM155 for 48 hours. Increased and dose dependent Caspase-3/7 activity was observed in NEC-DUE1 **C.** and -DUE2 **D.** cells when treated with YM155 for 2 hours. E, PARP cleavage became obvious in the more sensitive NEC-DUE2 cell line. Cells were treated at indicated YM155 concentrations for 24 hours. Protein lysates were separated by SDS-PAGE and blotted membranes detected with specific antibodies recognizing uncleaved as well as cleaved PARP (cPARP). Statistical significance was calculated by two-tailed nonparametric Mann-Whitney test (**indicates a p-value ≤0.01; *indicates a p-value ≤0.05).

To further assess the potency of survivin small molecule antagonists to induce apoptotic cell death in NEC cell lines, we concentrated on compound YM155 exhibiting the highest efficacy even in nanomolar concentrations. Incubation of NEC cells with YM155 induced a statistically significant dose dependent increase in apoptotic cells as well as caspase-3/7 activation that was again pronounced in NEC-DUE2 cells (Figure 4A-4D). In addition, PARP (Poly ADP-ribose polymerase) cleavage as a marker for apoptosis appeared only in YM155 treated NEC-DUE2 cells (Figure 4E).

### Small molecule YM155 exhibits a potent antitumor activity in GEP-NEC xenografts

**Figure 5:**
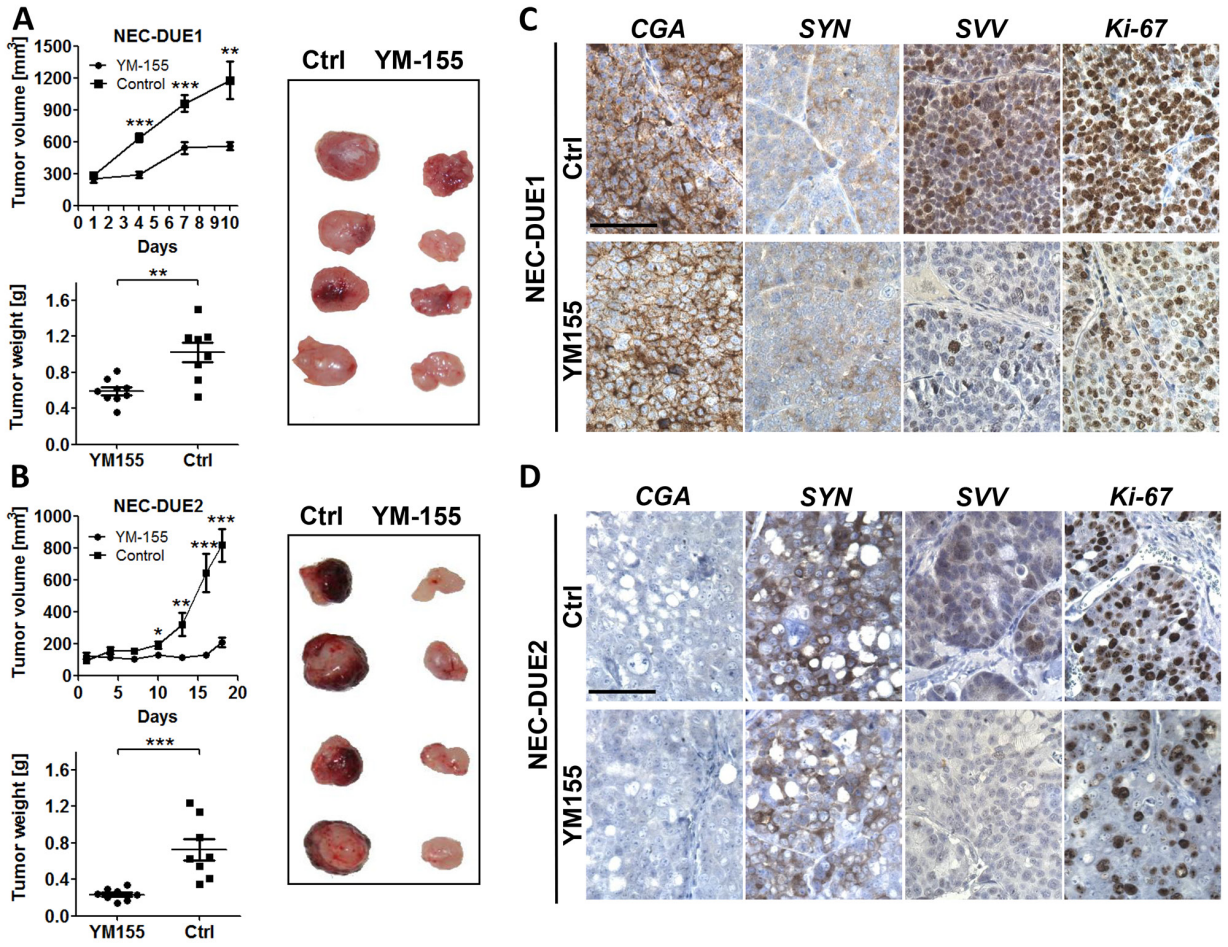
YM155 inhibits tumor growth in a GEP-NEC xenograft mouse model **A.** NOD-Scid IL2rgammanull mice bearing NEC-DUE1 xenografts were treated by daily injection of YM155 (3mg/kg) or vehicle only via intraperitoneal (i.p.) route. Tumor volumes were estimated twice weekly by two-dimensional caliper measurements.YM155 markedly impaired tumor growth (***indicates a p-value ≤0.001; **indicates a p-value ≤0.01; top graph). In addition, YM155 therapy significantly reduced tumor weight when comparing treatment with control group (**indicates a p-value ≤0.01; bottom graph). **B.** The experiment was repeated as described above using NEC-DUE2 xenografts. YM155 inhibited NEC-DUE2 tumor growth (***indicates a p-value ≤0.001; **indicates a p-value ≤0.01; *indicates a p-value ≤0.05). Tumor weight was significantly lower in YM155 treatment group when compared with control animals (***indicates a p-value ≤0.001). NEC-DUE2 showed an increased susceptibility to YM155 with a 3.1-fold reduction of tumor weight when compared to NEC-DUE1. Immunohistochemical staining of FFPE sections obtained from NEC-DUE1 **C.** and NEC-DUE2 **D.** xenografts verified a YM155 depending decrease in survivin protein levels when comparing controls (Ctrl) with teratment groups. General neuroendocrine markers chromogranin A (CgA) and synaptophysin (SYN) were unaffected by i.p. YM155 application. Proliferation was assessed by Ki-67 staining. Images were captured at 400 × magnification and scale bar indicates 25 µm.

In order to assess the effect of YM155 on the *in vivo* tumor growth of NEC cells, we produced localized tumors in a xenograft mouse model by injecting NEC-DUE1 or -DUE2 cells subcutaneously into the flank of immunocompromized mice. Daily i.p. administration of YM155 was initiated after tumor nodules reached a size of approximately 100-200 mm^3^. Control mice were treated via the same route with saline vehicle solution. Importantly, we did not observe any adverse effects and treatment was well tolerated. YM155 significantly abrogated tumor growth in both NEC-DUE1 and NEC-DUE2 xenograft models. The mean volume for NEC-DUE1 tumors at the study endpoint was 561.3 ± 38.24 mm^3^ for YM155 treated animals versus 1182 ± 173.6 mm^3^ for the control group (p = 0.008) (Figure 5A). Also, NEC-DUE2 tumors treated with YM155 formed smaller tumors (818.4 ± 101.0 mm^3^) when compared to control animals (210.2 ± 30.9 mm^3^) (p < 0.001) (Figure 5B). Of note, tumors formed by NEC-DUE1 cells reached the acceptable tumor volume faster than NEC-DUE2 tumors. Interestingly, NEC-DUE2 tumors showed an increased susceptibility to YM155 with a 3.1-fold reduction of tumor weight when compared to NEC-DUE1 treated tumors (1.7-fold reduction). Immunohistochemical staining of tissue sections obtained from tumors of each experiment confirmed a decrease of intratumoral survivin protein levels in YM155 treated mice (Figure 5C and 5D). In contrast, expression levels of general neuroendocrine markers chromogranin A and synaptophysin remained unchanged. Furthermore, expression of Ki-67 proliferation marker was decreased in YM155 treated NEC-DUE1 and -DUE2 tumors (Supplementary Figure 4A and 4B).

## DISCUSSION

Motivated by the urgent need to identify novel molecular targets that might be of potential therapeutic relevance in GEP-NEN, we focused on survivin which has been reported to be associated with poor prognosis in G1 and G2 GEP-NET, formerly categorized as well differentiated NET [31]. Since survivin interacts with IAP family member XIAP in several cellular processes such as inhibition of programmed cell death and activation of transcription factor NF-κB [13–15], we also aimed to unravel the potential role of XIAP in GEP-NEN tumor biology.

Our initial experiments, identifying a CNG of the BIRC5 locus in 60 % of the included GEP-NEN tissue specimens confirm the previously reported frequency of genetic gains on BIRC5 encoding chromosome 17q in 57 % of midgut NEN [30]. Interestingly, there was no difference in the frequency of BIRC5 CNAs when comparing the subgroup of GEP-NET with the biologically more aggressive NECs. Although XIAP CNAs were less frequent in GEP-NEN, a higher percentage of CNAs became evident in G3 NECs than in G1 and G2 NETs. The observation that survivin protein level did not correlate with the presence of BIRC5 copy number alterations suggests that other mechanism such as methylation, proteasomal degradation and transcriptional regulation might be involved to drive aberrant expression of survivin in GEP-NEN [33–36]. In this context, Vaira et al. demonstrated that insulin-like growth factor 1 (IGF-1), which is expressed in many NETs and involved in regulating the growth of NET cells via an autocrine route, induces the expression of survivin [36–38].

However, we observed significantly increased expression levels of nuclear and cytoplasmic survivin in G3 NECs with high mitotic index which, from the biological point of view, is in line with the observation that survivin plays a critical role during mitosis in cancer cells [39]. Thus, it is tempting to speculate that nuclear survivin as a chromosomal passenger molecule participates in an uncontrolled proliferation especially of highly proliferative GEP-NECs. Although univariate analysis identified both expression of survivin and proliferation based grading to be negatively associated with patients survival, multivariate analysis revealed only high grading as a predictive factor for overall survival. Thus, our multivariate analysis is potentially confounded by the interdependence between survivin as pro-proliferative factor and proliferation based grading per se. In contrast, we observed no correlation between XIAP expression levels and proliferation based grading or patients outcome.

In our functional experiments, we demonstrated an inhibitory effect on cell proliferation and tumor growth in NEC cells after knockdown of survivin. Furthermore, we found that small molecule YM155 reduced survivin protein expression in a dose dependent manner in NEC cell lines. This effect was accompanied by a decrease in cell viability, proliferation and an increase in apoptotic cell death. Importantly, the antitumor potency of YM155 was verified in a xenogratft mouse model of subcutaneously injected NEC-DUE1 and -DUE2 cells.

We also performed XIAP knock down experiments by using the shRNA approach or inhibitory studies incubating cells with small molecule XIAP antagonists GDC-0152 and Birinapant. Whereas shRNA mediated XIAP knock down reduced cell viability and tumor growth of NEC cells, small molecule smac mimetics failed to inhibit cell proliferation *in vitro* and were therefore not further validated in our *in vivo* mouse models. Mechanistically, recent data suggest that smac mimetics induce programmed cell death by directly binding XIAP to prevent the interaction with caspase-3, -7, and -9 [29]. In addition, smac mimetics induce rapid proteasomal degradation of cIAP1 and cIAP2 [40, 41]. Unfortunately, a low response rate to smac mimetics is a well known phenomenon being supported by the observation that most of the investigated cell lines are not or only moderately sensitive to these small molecule anatgonists [29]. Thus, our data demonstrating a sufficient antiproliferative effect upon genetic ablation of XIAP but not after treatment with smac mimetics might be explained by recent studies identifying mechansims that contribute to smac mimetic resistance in malignant cells. One such cell death evading mechanism involves a tumor necrosis factor α (TNFα) mediated up-regulation of cIAP2 via NF-κB [42]. Other factors that have been supposed to induce smac mimetic resistance involve a defective phosphatidylinositide 3-kinase (PI3K) signaling pathway or the inability to form a ripoptosome complex upon cIAP degradation [42, 43].

In addition to our ex vivo data demonstrating a grading-dependent overexpression of survivin in GEP-NEN, our study provides first evidence that survivin plays a crucial role in the tumorigenesis of GEP-NEC by regulating proliferation and apoptosis. This phenomenon has been described for a number of different tumor entities and led to an intensified search for potent molecular inhibitors that are suitable to selectively target survivin. So far, only a small number of pre-clinically tested antagonists including antisense oligonucleotides, peptidomimetics, dominant interfering mutants, immunotherapeutics or transcriptional repressors have entered first clinical trials [44]. One of the most extensively investigated survivin antagonists is the transcriptional repressor YM155. YM155 initially identified by a high-throughput screening inhibits Sp1-dependent survivin promotor activation resulting in a marked reduction of survivin on protein level [28, 45]. Importantly, YM155 produced impressive results in several experimental animal models using cancer cell lines originating from anaplastic thyroid cancer, soft tissue tumors, non-small-cell-lung cancer or prostate cancer and led ultimately to the evaluation of YM155 in multicenter Phase II clinical trials [26, 27, 45–47].

To date, chemotherapeutic agents recommended for the treatment of G3 NEC are primarily based on a combinational therapy with cisplatin and etoposide, but demonstrate only frustrating results with response rates of 50 % [9, 10]. Thus, our data fit perfectly to pre-clinical data investigating the efficiacy of anti-survivin therapies and might therefore fill the gap in identifying novel therapeutic targets particularly for the treatment of highly aggressive GEP-NEC that are urgently needed. Importantly, our data are very encouraging and might therefore justify the inclusion of patients with GEP-NEC into clinical trials using anti-survivin based therapies. However, future pre-clinical work will have to prove the efficiacy of a monotherapy with survivin antagonists or if combinational treatment regimes, as they are currently ongoing for other tumor entities, will enhance the antitumor effects of survivin antagonists also in GEP-NEN.

## MATERIALS AND METHODS

### Ethics statement

Investigation has been conducted in accordance with the ethical standards and according to the Declaration of Helsinki and according to national and international guidelines and has been approved by the authors’ institutional review board.

### Patient selection and clinicopathological data

A retrospective search of archived formalin-fixed and paraffin-embedded (FFPE) primary GEP-NEN tissue specimens at the Institute of Pathology (University Hospital Duesseldorf) revealed 84 patients who had undergone surgical resection between February 1998 and January 2013. Hematoxylin and Eosin (H&E)-, synaptophysin-, CgA and MIB-1-stained sections of all specimens were reviewed by a pathologist (JCR) to define the histological grade of the tumor and to mark representative tumor regions on H&E-microscope slides. After exclusion of tissue specimens with insufficient tumor material a total of 77 primary GEP-NEN were finally included in this study. Tumor stage and grading were determined as outlined by the 7^th^ edition of the World Health Organisation (WHO) [4]. Data on clinical parameters including age, sex, localization of the primary tumor, date of surgery, type of operative procedure and date of last follow up or death were reviewed. An institutional review board (IRB)-approval of the Medical Faculty, Heinrich Heine University Duesseldorf (IRB-No: 3821) was retrieved.

### Immunohistochemistry

Immunohistochemistry of tissue microarray (TMA) slides and xenograft derived tumor specimens was performed as recently described [18]. Tissue slides of pre-tested human tonsille and colon cancer, known to express survivin and XIAP, served as positive control. In addition, control sections were processed as described above with non-binding IgG, and resulted in no detectable staining. Expression levels of survivin and XIAP were estimated by two investigators (KO and LD) independently according to the immunoreactive score (IRS) ranging from 0 to 12 [48]. This score is calculated by multiplying the intensity of staining (0 = no staining; 1 = weak staining; 2 = strong staining; 3 = very strong staining) by the percentage of positive cells graded as 0% (0); <10% (1); 11–50% (2); 51–80% (3); 81–100%. For survivin, nuclear and cytoplasmatic protein expression were separately determined. An IRS > 2 was defined as high protein expression level whereas an IRS ≤ 2 as low protein expression.

### Cell culture and lentiviral transduction

GEP-NEC cell lines NEC-DUE1 and NEC-DUE2 have been established in our laboratory [49]. Both NEC cell lines were maintained in RPMI medium supplemented with 10% heat inactivated FCS, penicillin and streptomycin at 37°C in an atmosphere with 5% CO_2_. Authenticity and purity were confirmed by DNA fingerprinting as recently described [49].

Viral particles were obtained by transfection of HEK293FT using Lipofectamine2000 reagent with envelope plasmid pCMV-VSVG, packaging plasmid psPAX2 (both Addgene, Cambridge, MA, USA) and pGIPZ-shXIAP (clone V2LHS-94578; mature antisense sequence: TTACAAGTGACTAGATGTC), pGIPZ-shBIRC5 (clone V2LHS_262484; mature antisense sequence: TTCCTAAGACATTGCTAAG) or pGIPZ non-silencing lentiviral shRNA control plasmid (all Open Biosystems, Dharmacon, Lafayette, CO, USA) according to the manufacturer's instructions.

For viral infection 8 × 10^4^ NEC cells were seeded per well in 24 well plates and grown over night in a 10% CO_2_ incubator at 37°C. The next day, culture medium was removed and 600 µl of viral supernatant completed with 4 µg polybrene was added to the cells for 20 minutes. After centrifugation for 2 hours at 1200 rpm and 32°C, 600 µl of fresh culture medium was added and cells were incubated under standard conditions. The next day, transduction procedure was repeated and cells were cultivated for 48 hours under standard conditions followed by selection in culture medium containing puromycin at concentrations of 1 µg/ml or 2 µg/ml, respectively. Knockdown was confirmed on mRNA and protein levels using qPCR and western blotting.

### Cell viability and proliferation

Cell viability and proliferation assays were performed with 5 × 10^4^ cells seeded per well in 96 well plates. Four days after incubation with YM155, M4N, Birinapant, GDC-0152 or DMSO as vehicle control at equimolar concentrations, cell viability was measured using the CellTiter 96® AQueous One Solution Cell Proliferation Assay (Promega, Madison, WI, USA) and proliferation was quantified based on the BrdU-incorporation (Colorimetric Cell Proliferation ELISA; Roche Life Science, Indianapolis, IN, USA) according to the manufacturer's recommandations. Assays were carried out in triplicates and the mean IC_50_ was obtained based on the results of 3 independent experiments.

### Cell death assays

Annexin-V/PI staining and FACS analysis was performed to measure induction of apoptosis after treatment of 5×10^5^ NEC-DUE1 or NEC-DUE2 cells seeded in 6-well plates with varying concentrations of YM155 or DMSO (vehicle control) for 48 hours by using the FITC Annexin V/Dead Cell Apoptosis Kit (Molecular Probes, Eugene, OR, USA) according to the manufacturere's protocol.

Caspase activity was determined using the Caspase-Glo3/7 luminescent assay (Promega Madison, WI, USA) according to the manufacturer's instructions. Therefore, 5×10^4^ cells per well were seeded in 96 well plates and treated with varying concentrations of YM155 or vehicle control (DMSO) for 2 hours. The luminescence of each sample was determined using the Infinite® 200 PRO microplate reader (Tecan Group Ltd., Crailsheim, Germany). Luminescence signals of drug treated cells were normalized to those of control cells. The mean results were obtained from triplicates.

### *In vivo* animal models

All animal procedures were evaluated and approved by the North-Rhine-Westfalian (NRW) Ministry for Environment and Nature Protection, Agriculture and Consumer Protection (Landesamt für Natur, Umwelt und Verbraucherschutz; LANUV NRW: 84-02.04.2014.A208). For experiments evaluating the effect of a stable shRNA knockdown of survivin or XIAP on local tumor growth, 1×10^6^ NEC-DUE1 or NEC-DUE2 gene specific knockdown cells were resolved in 200 μl sterile Matrigel/PBS solution and subcutaneously injected into the left flank region of 6-8-week-old NOD-Scid IL2rgamma^null^ mice. NEC-DUE1 or -DUE2 cells transduced with unspecific shRNA were injected in the other side, so mice could serve as their own control. Three to five weeks after injection mice were sacrificed.

To evaluate the effect of a pharmacological survivin inhibition on tumor growth in GEP-NEC cell lines *in vivo* we used the small-molecule antagonist YM155. Therefore, 1×10^6^ NEC-DUE1 or NEC-DUE2 cells were injected subcutaneously into the left flank of 6-8-week-old NOD-Scid IL2rgamma^null^ mice (n = 9/treatment group). After establishment of palpable nodules, tumor volumes were estimated twice weekly by two-dimensional caliper measurements using the equation v = (l×w^2^/2) [v = volume (mm^3^); l = length (mm), w = width (mm)]. When tumors reached 100 mm^3^ to 200 mm^3^, animals were allocated to two groups, so that the mean tumor volume in each group was comparable, and treated with YM155 (3mg/kg/d) or sterile saline vehicle solution by daily intraperitoneal gavage. Mice were sacrificed when control group tumors reached a volume of approximately 1200 mm^3^. Tumors were carefully removed, measured, weighed, fixed in formalin and paraffin embedded for immunohistochemical analysis.

### Statistical analysis

Differences in the DNA copy number status (gain versus no gain) or protein expression levels (high versus low) according to clinicopathological variables were examined using the Chi square test. Overall survival was defined as the period from the date of surgery until death or until the date of the last follow up at which survivors were censored. Patients who died within 30 days of operation or had incomplete tumor resections were excluded from the survival analysis. For survival analysis, Kaplan-Meier curves were generated and assessed using the log-rank (Mantel Cox) test. For multivariate survival analysis all variables were included into a Cox regression analysis. Results are presented as hazard ratio (HR) with 95% confidence intervals (CI).

Differences in tumor size and weight of xenografts were analyzed by Wilcoxon matched pairs test for knock down experiments and by two-tailed Mann-Whitney test for YM-155 treatment experiments. Cell culture-based quantitative assays were repeated at least 3 times and mean ± standard error of mean (SEM), median and range were calculated and assesed for statistical significance by a two-tailed nonparametric Mann-Whitney test. The mean 50% inhibitory concentration (IC_50_) was calculated by logistic analysis. All statistical analyses were performed using GraphPad Prism for Windows (Version 5, GraphPad Software, San Diego, California, USA) or SPSS statistics for Windows (Version17.0, Chicago: SPSS Inc.). P-values < 0.05 were considered statistically significant.

## SUPPLEMENTARY MATERIALS FIGURES AND TABLES


