# Supplementary material for: Preclinical assesement of survivin and XIAP as prognostic biomarkers and therapeutic targets in gastroenteropancreatic neuroendocrine neoplasia
Source: Oncotarget. 2016 Dec 26;8(5):8369–82. doi: 10.18632/oncotarget.14207 (PMC5352407; doi:10.18632/oncotarget.14207)
Supplement: Supplementary file 1 [file oncotarget-08-8369-s001.pdf]

Preclinical assesement of survivin and XIAP as prognostic biomarkers and therapeutic targets in gastroenteropancreatic neuroendocrine neoplasia

SUPPLEMENTARY MATERIALS AND METHODS

Reagents

Sepantronium Bromide (YM155), Birinapant (TL32711) as well as GDC-0152 were purchased from Selleckchem (Houston, TX, USA) and Tetra-O-methyl nordihydroguaiaretic acid (Terameprocol, M4N) from Sigma-Aldrich (St. Louis, MO, USA). For *in vitro* applications, all drugs were dissolved in dimethyl sulfoxid (DMSO). For *in vivo* animal experiments, YM155 was freshly prepared in sterile saline at a final concentration of 0.3 mg/ml. Antibodies selected for immunodetection by immunohistochemistry (IHC) or Western Blot (WB) analysis were raised against survivin (NB500-201; 1:750 dilution for IHC and 1:1000 dilution for WB, Novus, Littleton, CO, USA), XIAP (Clone 28, 1:1000 dilution for WB or 48,1:35 dilution for IHC; both BD Biosciences, San Jose, CA, USA), PARP (9542; 1:1000 dilution, Cell Signaling, Denver, MA, USA), Chromogranin A (MAB 5268; 1:1000 dilution, Chemikon, Schwalbach, Germany), Synaptophysin NCL-L-Synap 299; 1:100 dilution, Novocastra, Berlin, Germany), Ki-67 (Clone MIB-1; 1:500 dilution, Dako, Hamburg, Germany), cleaved caspase-3 (Asp175; 9661, 1:300 dilution, Cell Signaling), alpha-tubulin (Clone DM1A; 1:5000 dilution, Sigma-Aldrich), GAPDH (Clone 6C5; 1:5000 dilution, abcam, Cambridge, United Kingdom).

Tissue microarray

For tissue microarry (TMA) construction, representative tumor areas were microscopically selected and marked on H&E-stained sections by a pathologist (JCR). Marked slides were overlaid on the original FFPE block to identify the corresponding tumor area. Two representative tissue cores each of 1.0mm diameter were then extracted from the original tissue block and transferred into a recipient block using the Manual Tissue Arrayer MTA-1 (Beecher Instruments, Sun Prairie, WI, USA). Two cores of normal tonsil tissue and two cylinders from colon cancer specimen that served as control for immunohistochemical reaction were inserted into the right upper corner of each recipient block for orientation when examining the slides.

Immunoblotting

Cells were lysed in RIPA buffer (Sigma) supplemented with protease inhibitor mix (cOmplete, Roche Life Science, Indianapolis, IN, USA). Clarified lysates (20 µg) were boiled in Laemmli buffer, separated on SDS-PAGE gels and transferred to nitrocellulose membranes. After blocking with TBS-T buffer containing 2.5-5% nonfat dry milk, membranes were incubated overnight at 4°C with various antibodies as specifically indicated. Washed membranes were then incubated with horseradish peroxidase-conjugated secondary antibodies for 1 hour at room temperature and visualized by using the Immune-Star™ Western C™ Kit (BioRad, Hercules, CA, USA) with the Versa Doc Imaging System (BioRad).

RNA isolation and quantitative RT-PCR analysis

Total RNA from cell lines was isolated using the RNeasy Mini Kit (Qiagen, Hilden, Germany) according to the manufacturer´s instructions. Reverse transcription and quantitaive PCR were performed as described previously (1). Primer sequences and Taq Man Probes (Universal ProbeLibrary Set human, Roche) were as follows: for survivin (Gene accession number: NM_001168.2) forward primer, GCCCAGTGTTTCTTCTGCTT and reverse primer, AACCGGACGAATGCTTTTTA, probe No. 11; for XIAP (Gene accession number: NM_001167) forward primer, ACTTTTGGGACATGGATATACTCAG and reverse primer, AGCACTTTACTTTATCACCTTCACC, probe No. 68; GAPDH forward primer, AGCCACAT CGCTCAGACAC and reverse primer, GCCCAATACGA CCAAATCC, probe No. 60. Gene expression was quantified by using the $2^{-\Delta\Delta CT}$ method (2). Specificity of PCR products was controlled by sequencencing with gene specific oligonucleotides.

DNA Preparation and Comparative Genomic Hybridization with Oligonucleotide Microarrays (aCGH)

Genomic DNA was isolated from FFPE tissue specimens of primary tumors and Copy number alterations (CNA) were analyzed by performing aCGH analyses on an oligonucleotide-based platform (Agilent Oligonucleotide Array-Based CGH for Genomic DNA Analysis, Version 7.1; Agilent Technologies, Santa Clara, CA, USA) as recently described (3). Sex matched gDNA from normal duodenal mucosa served as reference.

REFERENCES

1. Luca AC, Mersch S, Deenen R, Schmidt S, Messner I, Schafer KL, et al. Impact of the 3D microenvironment on phenotype, gene expression, and EGFR inhibition of colorectal cancer cell lines. PLoS One.8:e59689.
2. Livak KJ, Schmittgen TD. Analysis of relative gene expression data using real-time quantitative PCR and the 2(-Delta Delta C(T)) Method. Methods. 2001;25:402-8. Epub 2002/02/16.
3. Mersch S, Riemer JC, Schlunder PM, Ghadimi MP, Ashmawy H, Mohlendick B, et al. Peritoneal sarcomatosis: site of origin for the establishment of an *in vitro* and *in vivo* cell line model to study therapeutic resistance in dedifferentiated liposarcoma. Tumour biology : the journal of the International Society for Oncodevelopmental Biology and Medicine. 2015. Epub 2015/09/17.

SUPPLEMENTARY FIGURES AND TABLES

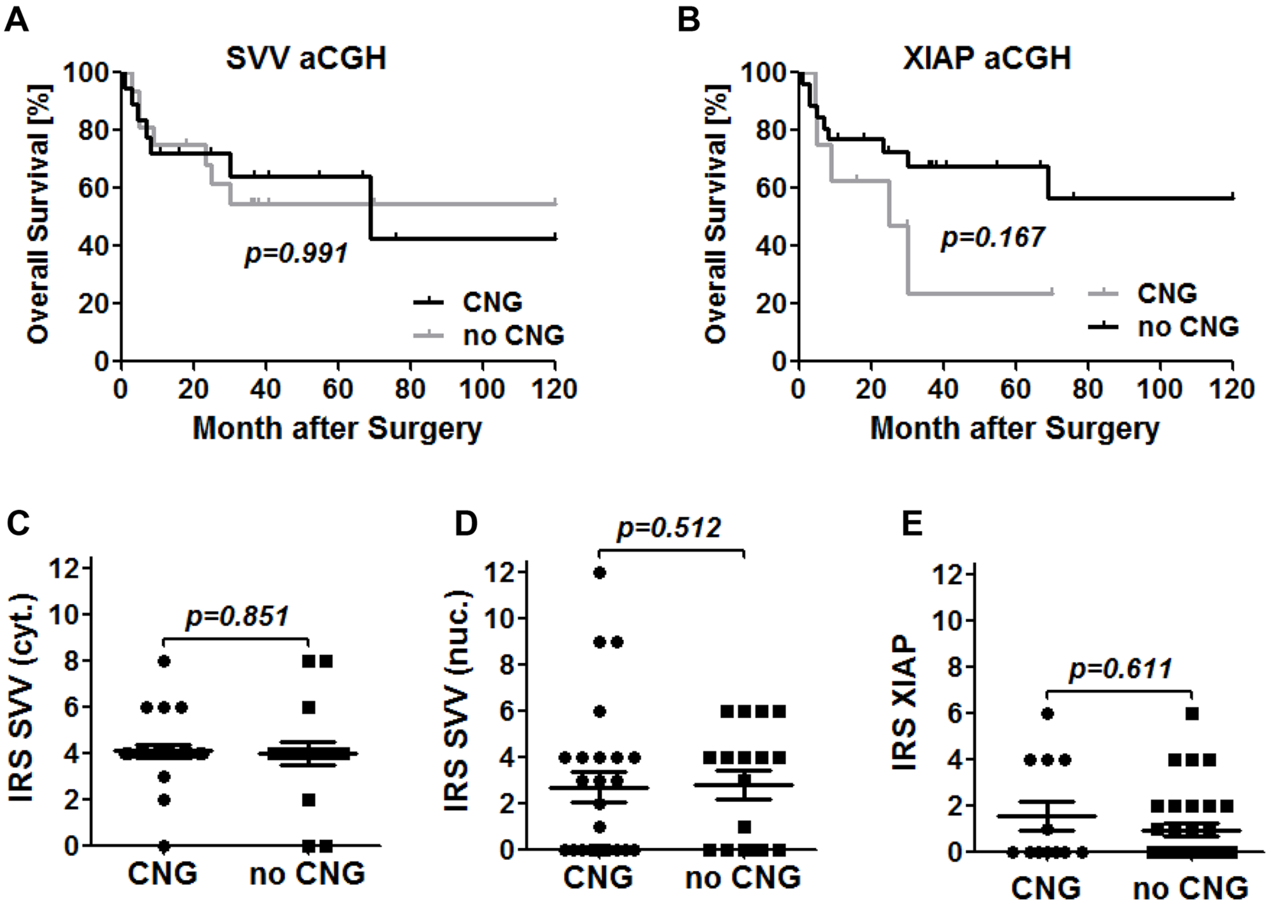

Supplementary Figure 1: Copy number gains at the survivin and XIAP gene locus are not associated with overall survival and protein expression levels. Groups of patients with copy number gains (CNG) or without CNGs at the **A.** survivin locus and **B.** XIAP locus were analyzed for overall survival using Kaplan-Meier curves. Protein expression levels of **C.** cytoplasmic and **D.** nuclear survivin as well as **E.** XIAP were not associated with CNGs of included patients with GEP-NEN.

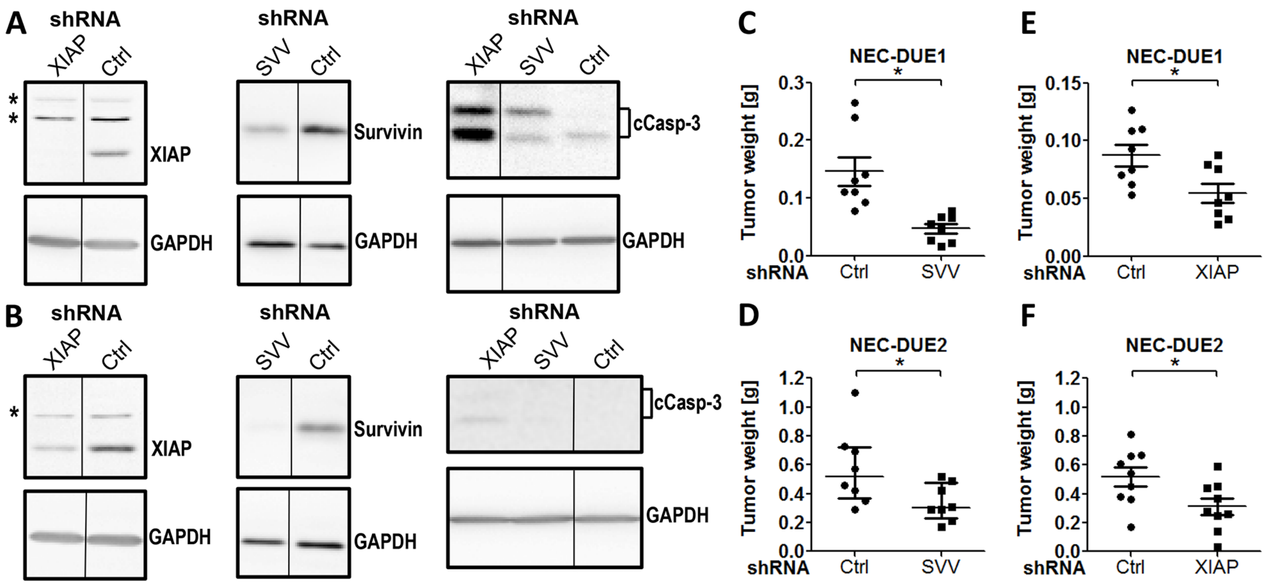

Supplementary Figure 2: Gene-specific knockdown of survivin or XIAP activates caspase-3 and abrogates tumor weight of GEP-NEC xenografts. Caspase-3 cleavage (cCasp-3) was detected by Western Blot analysis in XIAP and survivin knockdown or control (Ctrl) NEC-DUE1 **A.** and NEC-DUE2 **B.** cells as indicated. Dividing vertical lines indicate that individual lanes were combined from a single electrophoresis gel and * indicates unspecific bands. NEC-DUE1 **C.** and **E.** or NEC-DUE2 **D.** and **F.** cells transduced with gene specific shRNAs targeting survivin (C and D) or XIAP (E and F) formed tumors with a reduced weight when compared to NEC cells transduced with an unspecific control (Ctrl) shRNA. Values are expressed in means ± SEM of at least 8 tumors per shRNA transduced and injected cell line. Statistical significance was calculated by Wilcoxon matched pairs test (*indicates a p-value ≤0.05).

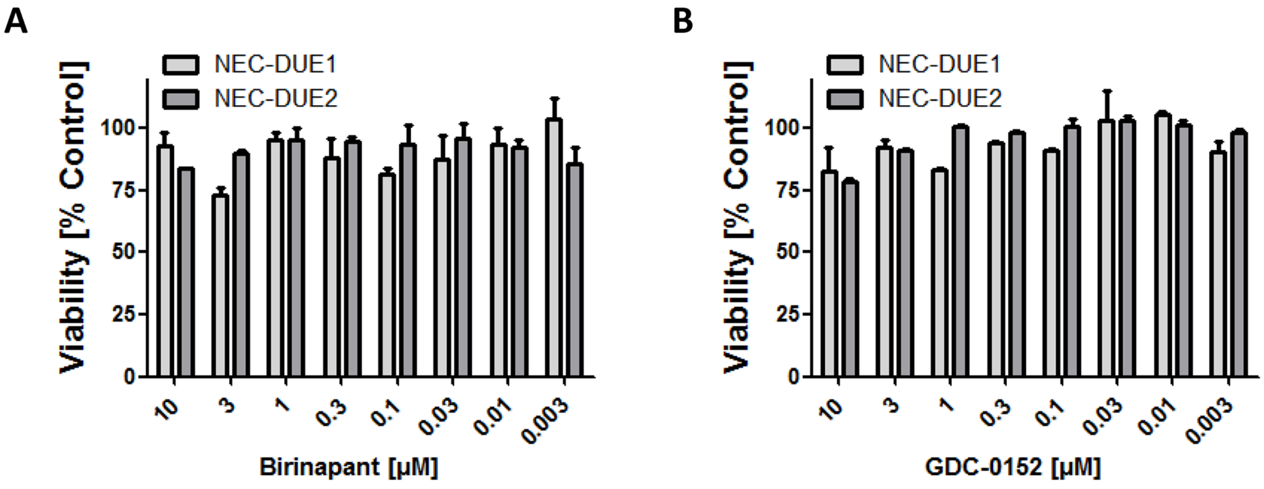

Supplementary Figure 3: XIAP small molecule antagonists fail to significantly reduce cell viability in GEP-NEC cell lines. 5×10^4 cells were seeded per well in 96 well plates and incubated for 4 days with increasing concentrations of **A.** Birinapant or **B.** GDC-0152 as indicated. DMSO served as vehicle control at equimolar concentrations. MTS assays were performed in triplicates.

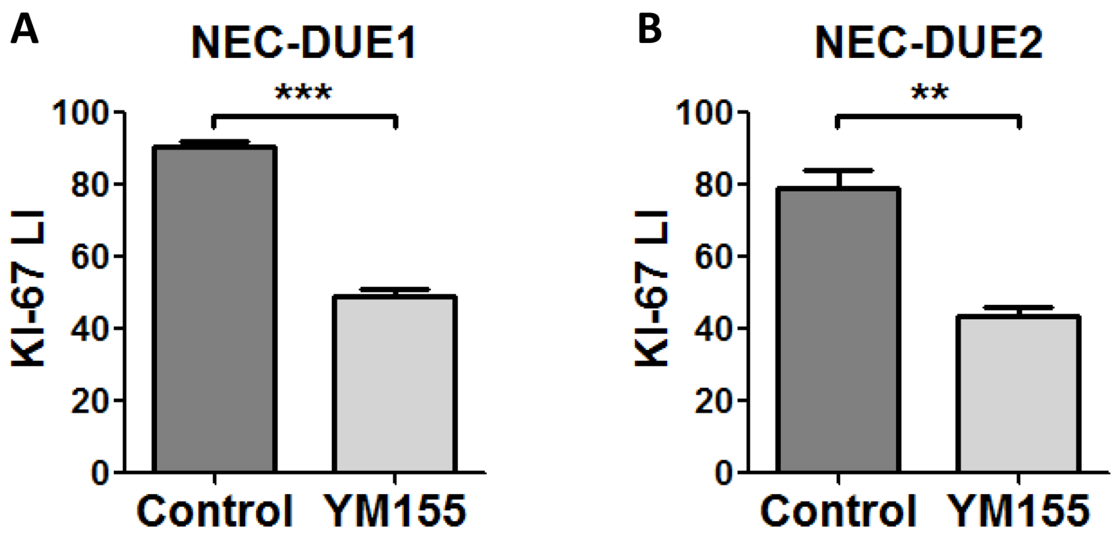

Supplementary Figure 4: YM155 decreases expression of Ki-67 proliferation marker in GEP-NEC tumor nodules. Immunohistochemical staining of FFPE sections obtained from NEC-DUE1 and NEC-DUE2 xenografts verified a YM155 dependent decrease in Ki-67 labeling index (Ki-67 LI) when comparing control group tumors with treatment group tumors. **A.** Mean Ki-67 LI for NEC-DUE1 control group tumors was 90.5% versus 49.1% for the YM155 treated tumors ($p < 0.001$). **B.** Mean Ki-67 LI for NEC-DUE2 control group tumors was 79.3% versus 43.5% for the YM155 treated tumors ($p = 0.003$). Values are expressed in means ± SEM. Statistical significance was calculated by unpaired T-test (**indicates a p-value ≤0.01; *** indicates a p-value ≤0.001).

Supplementary Table 1: Patient characteristics

Variable	No. of Patients (%)
Age	
≤ 65	35 (45.5)
> 65	41 (53.2)
Unknown	1 (1.3)
Sex	
Male	38 (49.4)
Female	39 (50.6)
T-category	
T1	13 (16.9)
T2	19 (24.7)
T3	29 (37.7)
T4	10 (13)
Unknown	6 (7.8)
Lymph node metastasis	
Negative	33 (42.9)
Positive	39 (50.6)
Unknown	5 (6.5)
Distant metastasis	
Negative	50 (64.9)
Positive	27 (35.1)
Grading	
G1	46 (59.7)
G2	11 (14.3)
G3	20 (26)
Resection margins	
Negative	65 (84.4)
Positive	12 (15.6)
Localisation	
Pancreas	35 (45.5)
Stomach	9 (11.7)
Small intestine	15 (19.5)
Colon	18 (23.4)

Supplementary Table 2: Overall Survival: Univariate analysis

Variable	Patients (n)	HR	95% CI (lower-upper)	*p*-value
**Age**				
≤ median	28	1		
> median	29	2.524	0.854 - 7.464	0.082
**Sex**				
Female	28	1		
Male	29	0.789	0.280 - 2.221	0.652
**T- category**				
T1-T2	25	1		
T3-T4	29	3.622	0.992 - 13.215	**0.036**
**Lymph nodes**				
N0	30	1		
N+	26	7.711	1.725 - 34.470	**0.001**
**Grading**				
G1-G2	43	1		
G3	14	9.585	2.909 - 31.585	**< 0.001**
**Metastasis**				
M0	42	1		
M+	15	3.001	1.085 - 8.302	**0.026**
**Survivin expression (nuc.)**				
IRS ≤ 2	35	1		
IRS > 2	22	4.519	1.526 - 13.387	**0.003**
**Survivin expression (cyt.)**				
IRS ≤ 2	11	1		
IRS > 2	46	29.210	0.139 - open	**0.045**
**XIAP expression**				
IRS ≤ 2	49	1		
IRS >2	8	1.245	0.277 - 5.600	0.774

Supplementary Table 3: Overall Survival: Multivariate analysis

Variable	Patients (n)	HR	95% CI (lower-upper)	*p*-value
Grading				
G1-G2	43	1		
G3	14	5.211	1.457 – 18.636	0.011
Lymph nodes				
N0	30	1		
N+	26	11.704	1.486 - 92.156	0.019
